# Ear Acupuncture Therapy for Masticatory Myofascial and Temporomandibular Pain: A Controlled Clinical Trial

**DOI:** 10.1155/2015/342507

**Published:** 2015-08-17

**Authors:** Luciano Ambrosio Ferreira, Eduardo Grossmann, Eduardo Januzzi, Rafael Tardin Rosa Ferraz Gonçalves, Fernando Antonio Guedes Mares, Marcos Vinicius Queiroz de Paula, Antonio Carlos Pires Carvalho

**Affiliations:** ^1^Departamento de Radiologia da Faculdade de Medicina, Universidade Federal do Rio de Janeiro, Rua Luiz Andrade Silveira 207, Centenário, 36045-280 Juiz de Fora, MG, Brazil; ^2^Universidade Federal do Rio Grande do Sul, Rua Coronel Corte Real 513, Petrópolis, 90630-080 Porto Alegre, RS, Brazil; ^3^Facsete/Ciodonto Faculdade de Tecnologia de Sete Lagoas, Avenida Prudente Morais 287, Sala 703, 30350-093 Belo Horizonte, MG, Brazil; ^4^Facsete/Ciodonto Faculdade de Tecnologia de Sete Lagoas, Rua Padre Marinho 98, Santa Efigênia, 30140-040 Belo Horizonte, MG, Brazil; ^5^Escola Paulista de Medicina, Unifesp, Avenida Brasil 1438, Sala 906, Funcionários, 30140-003 Belo Horizonte, MG, Brazil; ^6^Faculdade de Odontologia, Universidade Federal de Juiz de Fora (UFJF), s/n, Martelos, 36036-330 Juiz de Fora, MG, Brazil; ^7^Departamento de Radiologia da Faculdade de Medicina, Universidade Federal do Rio de Janeiro, CCS, Avenida Carlos Chagas Filho 373, Bloco K, 2° Andar, Sala 49, Cidade Universitária, Ilha do Fundão, 21941-902 Rio de Janeiro, RJ, Brazil

## Abstract

Ear acupuncture works by reducing painful sensations with analgesic effect through microsystem therapy and has been demonstrated to be as effective as conventional therapies in the control of facial pain. This clinical trial aimed to evaluate the adjuvant action of auricular acupuncture through an observation of the evolution of temporomandibular and masticatory myofascial symptoms in two groups defined by the therapies elected: auricular acupuncture associated with occlusal splint (study) and the use of the occlusal splint plate alone (control). We have selected 20 patients, who were randomly allocated into two groups of ten individuals. Symptoms were evaluated in five different moments, every seven days. We analyzed the orofacial muscle and joint palpation in order to measure the intensity of the experienced pain. Both groups showed a statistically significant decrease in muscle and joint symptoms (*p* < 0.05). However, comparisons between the groups showed an expressive and significant reduction of symptomatology in the study group (*p* < 0.05) already on the first week of therapy. According to the results, to the methodological criteria developed and statistical analysis applied, the conclusion is that auricular acupuncture therapy has synergistic action on conventional occlusal splint treatment. It was demonstrated to be effective in the reduction of symptoms in the short term.

## 1. Introduction

*Temporomandibular Disorder (TMD)*. It is a conjunct disease that affects masticatory muscles, temporomandibular joint (TMJ), teeth, and periodontal and orofacial associated structures [1, 2]. Inflammatory and infectious disturbances, trauma, and hormonal changes have been cited as common causes for TMD [1, 3, 4]. It is often associated with parafunctional habits and psychosocial disorders [2, 3]. Most of the authors consider the interaction of all risk factors, characterizing it as a multifactorial etiology [1, 3, 4].

TMD has demonstrated severe symptomatic manifestation in 2–10% of the population, most of it in women (83.7%) [5–7]. Among the main complaints, patients have reported masticatory myalgia and arthralgia [1–4, 6]. The most common dentistry therapy is the occlusal splint, which aims for muscle-joint stability and function [8, 9]. It is considered to be a conservative and valuable approach in the remission of TMD symptoms, often being associated with adjuvant therapies, such as pharmacological ones [10]. However, we have found an excessive use of allopathic medication, self-medication, or prescription in TMD patients, leading to frequent side effects and adverse reactions [11].

*Acupuncture Analgesia/Ear Acupuncture*. Acupuncture analgesia has been highly remarked in scientific research on acute and chronic disorder studies [12–14]. Western medicine explains acupuncture analgesia through the blocking of painful stimulus, which has proven to be as effective as conventional therapies [14] in the control of facial pain. Its use has been justified by slow onset of analgesia and long-lasting results with cumulative effect [5, 15, 16]. Scientifically, this occurs by nociceptors' synapses blockage over the central and peripheral nervous systems [17, 18]. The local fiber stimulation liberates cortisol, endorphins, dopamine, norepinephrine, and serotonin [18] through the activation of specific neural centers: the spinal cord, midbrain, and pituitary gland [19].

Ear acupuncture is an important part of the Traditional Chinese Medicine (TCM) [20], based on ancient concepts that consider that the activity of all organs and viscera, as well as their diseases, is manifested in the ear, as a reflex. Therefore, it is possible to analyze, evaluate, and treat morbid states by ear stimuli [21].

According to Wang [22], the tendinomuscular channels of the foot's* Yang Ming*, hand's* Tai Yang*, and the hand's* Shao Yang* are closely related to the outer ear. In addition, all three* Yang* channels of hands and feet are closely related to the ear in their path; by turns, the* Yin* channels relate to one another through different channels [21, 22]. Analgesic action at distance would be possible by physiological* Qi* and* Xue* movement. According to Gonzáles Garcia [21], occlusion of these channels would cause pain, while their clearance would produce analgesia.

Several studies have evaluated acupuncture therapeutic approaches aimed at relieving facial symptoms. They consider acupuncture as a simple, potentially effective, and useful technique for TMD treatment [14]. They have also reported it to be a valuable tool, complementary to conventional treatments [23], with immediate effects on the reduction of spontaneous pain and muscle tenderness [24], mainly when associated with occlusal splint [5, 25, 26]. As an adjuvant therapy, acupuncture was effective in the elimination of mouth opening restriction and in the control of muscle pain, with no complications, which demonstrates its safety [26].

Few studies describe the specific analgesic effect of the ear acupuncture technique. One of them [20] uses laser stimulus in ear points:* Shen Men*,* handle*,* lung*, and* dermis* to assess the pain threshold. Thus, patients who have received the actual therapy showed positive results in comparison with those in the placebo group.

Studies report the analgesic efficacy of traditional acupuncture in the treatment of chronic orofacial pain. However, studies on ear acupuncture that consider this specific theme are scarce. This study aims to evaluate the evolution of painful symptoms within a month, in women with TMJ and masticatory muscle pain, who have been submitted to adjuvant ear acupuncture together with occlusal splint treatment, and compare their results with those of patients who have been treated only with occlusal splint. Furthermore, it aims to assess which group of patients was able to have their pain reduced more rapidly and which one had better results at the end of interventions.

## 2. Materials and Methods

This study was approved by the Research Ethics Committee of the Federal University of Juiz de Fora under number 1456.147.2008.

Twenty women were selected from the School of Dentistry of the Federal University of Juiz de Fora without distinction of race, ages between 18 and 56. The volunteers have been selected and submitted to therapy from March to June 2012. They agreed to participate in the study and signed the consent form.

Patients had been diagnosed with TMD through RDC/TMD and selected after palpation of the TMJ and masticatory muscles [27]. The patients chosen had been diagnosed at least 6 months ago, considering the first time they reported pain, had painful symptoms in at least four orofacial structures, and had reported centric and/or eccentric bruxism. The exclusion criteria were the following: orthodontic treatment; facial trauma history; pregnancy; acuphobia; use of analgesic/nonsteroidal anti-inflammatory drugs; and other support therapeutic modalities as psychotherapy, physical therapy, and speech therapy. Patients who were making use of self-medication were advised not to take those drugs during the study.

**Figure 1:**
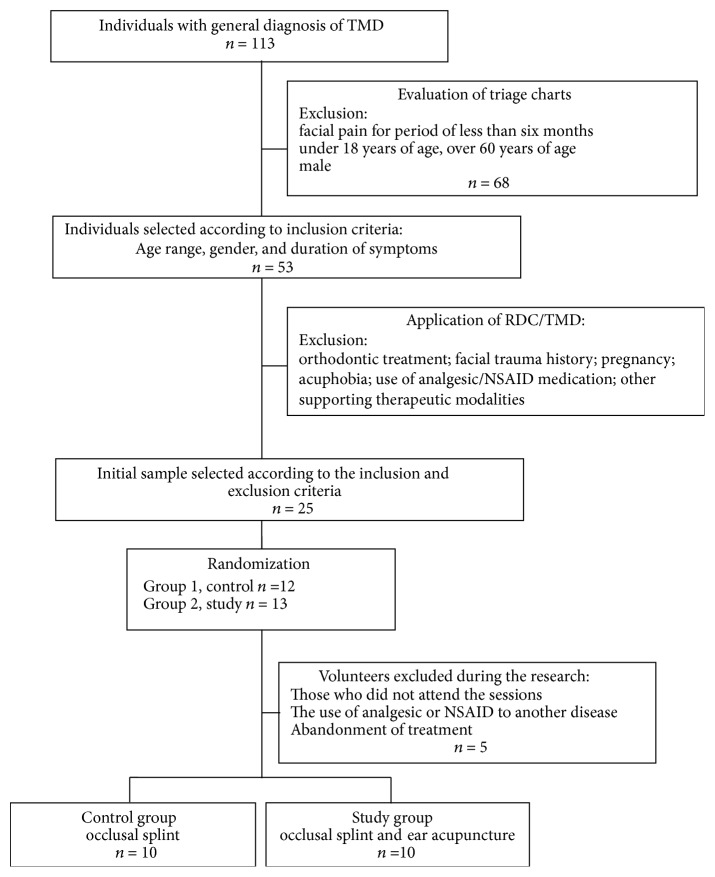
Trial profile.

The 20 patients were randomly divided into two groups: G1 (control), with ten patients who were treated exclusively with the occlusal splint; G2 (study) also composed of ten patients who were treated with auricular acupuncture associated with the occlusal splint (Figure 1).

**Figure 2:**
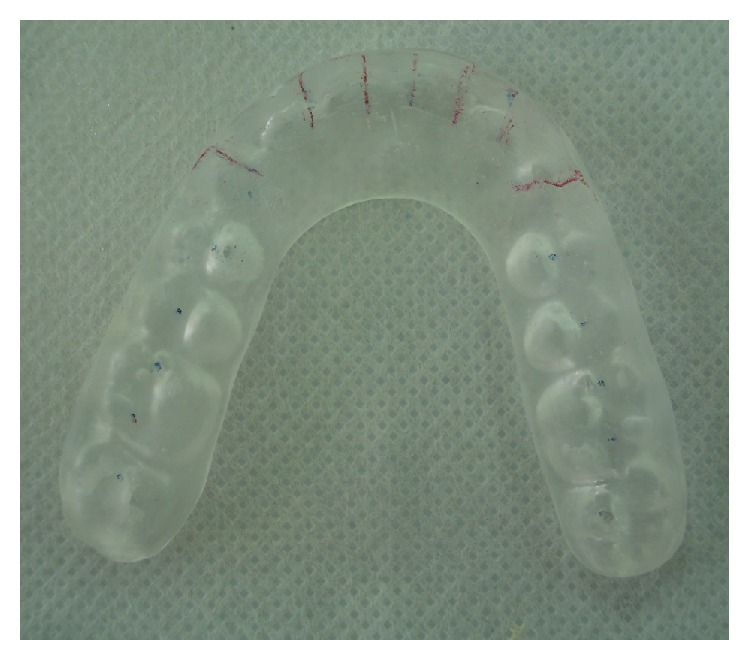
Occlusal splint (dental conventional therapy to bruxism).

**Figure 3:**
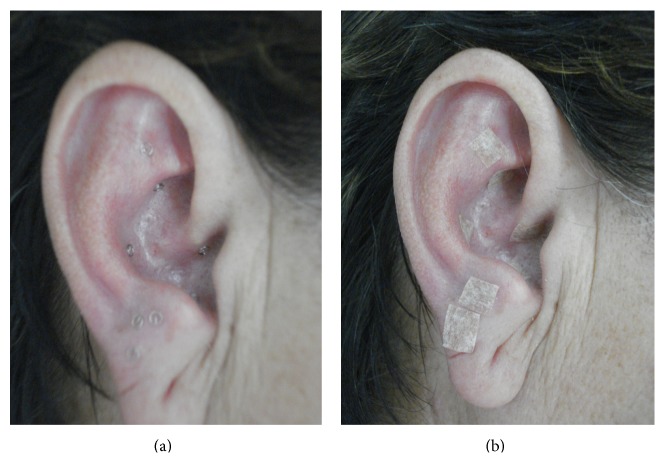
Ear acupuncture: (a) needles inserted into acupoints; (b) protection with hypoallergenic tape.

The same dentist was responsible for the occlusal adjustment on the acrylic splints (Figure 2) of both groups and for the weekly follow-up. The splint was used during nighttime sleep. The G2 patients received ear acupuncture in addition to the splint occlusal treatment, as showed in Figures 3(a) and 3(b). Therefore, the same acupuncturist used the electroacupuncture locator and stimulator therapy (EL30, NKL Ltd., Brusque, SC, Brazil) and intradermal needles of 1.0 mm in the ear region. The areas selected were those related to analgesia and to the systems of the orofacial structures affected. They received the microsystem technique [21, 28, 29]. Ear skin was cleaned with alcohol 70% and the ear channel was protected with dry cotton to prevent accidental needle insertion. The therapeutic approach was held weekly, in 5 sessions. Each session took about 50 minutes. During the session, the occlusal splint was checked and adjusted, retained needles were removed, and new needles were inserted and secured in the antagonist ear. The retention of needles in each ear lasted 5 days, on average.

We adopted the following ear points:* Shen Men* (in the triangular fossa ear, with analgesic and sedative properties);* Mouth* (under the root of helix, indicated for treatment of bucofacial conditions);* Kidney* (in the upper region of the Cymba shell, with regulatory properties of the nervous system and osteoarticular system, also treating tinnitus and arthralgia);* Liver* (in the lower region of Cymba, indicated for the treatment of diseases affecting muscles, ligaments, and tendons, also working as an analgesic and antispasmodic);* Spleen* (in the upper region of the cavum, indicated for disorders of the digestive system, including the mouth, where it opens, besides manifestations involving muscle activity and quality);* Maxillary* and* Jaw* regions (lobule, indicated for treating disorders of maxillofacial region); and* San Jiao* (antitragus region, indicated for the treatment of facial spasm, facial pain, and tinnitus) [21, 28, 29] (Figure 3).

After localization, there was an electrical stimulation of sedation or toning of the corresponding areas in one of the ears. We considered physical symptoms during Traditional Chinese Medicine (TCM) anamnesis and clinical signs observed by physical examination, pulse study, and tong evaluation, based on the eight-principle diagnostic technique,* Ba Gan Bian Zheng *[22, 28, 29].

After electrostimulation, intradermal needles were inserted into the antagonist ear and secured with hypoallergenic tape. Needle retention lasted 5–7 days. Each session, the right and the left ear were alternately submitted to electrostimulation and needle insertion. The volunteers were warned about adverse effects, as* De Qi* sensation during electrical stimulus or the sensation of the touch of the needle.

Each week, patients in both groups were submitted to a subjective assessment of pain, with a Visual Analog Scale (VAS) after palpation of orofacial structures. Patients were asked about the intensity of the pain, “10” being the maximum level of bearable pain and “0” being the absence of pain. The same blinded examiner performed muscle (medial and lateral pterygoid, masseter, and temporal muscle) and joint (retrodiscal and lateral pole of the condyle) assessment, applying 2.0 kg force in each muscle and 1.5 kg in the joints [27]. For this examination, we used a precision scale, with the proper calibration and previous training of the examiner. The evolution of symptoms in each area was measured by the mean scores of pain intensity for each muscle and joint in both groups.

*Statistical Analysis*. The evolution of symptoms, as well as the effectiveness of each therapy, was verified by an intragroup evaluation, with the submission of the results of the average VAS to an analysis of variance (nonparametric Friedman test), in order to verify if the values obtained during the period of treatment were statistically distinct. In order to compare both groups, we used the independent variables test (nonparametric Mann-Whitney). The significance value was 5%.

## 3. Results

The distribution of volunteers in each group, according to baselines variables, is demonstrated in Table 1.

The evaluation of symptoms during anamnesis by TCM with clinical signs observed by physical examination, pulse study, and tong evaluation showed the initial eastern diagnosis of patients (Table 2).

**Figure 4:**
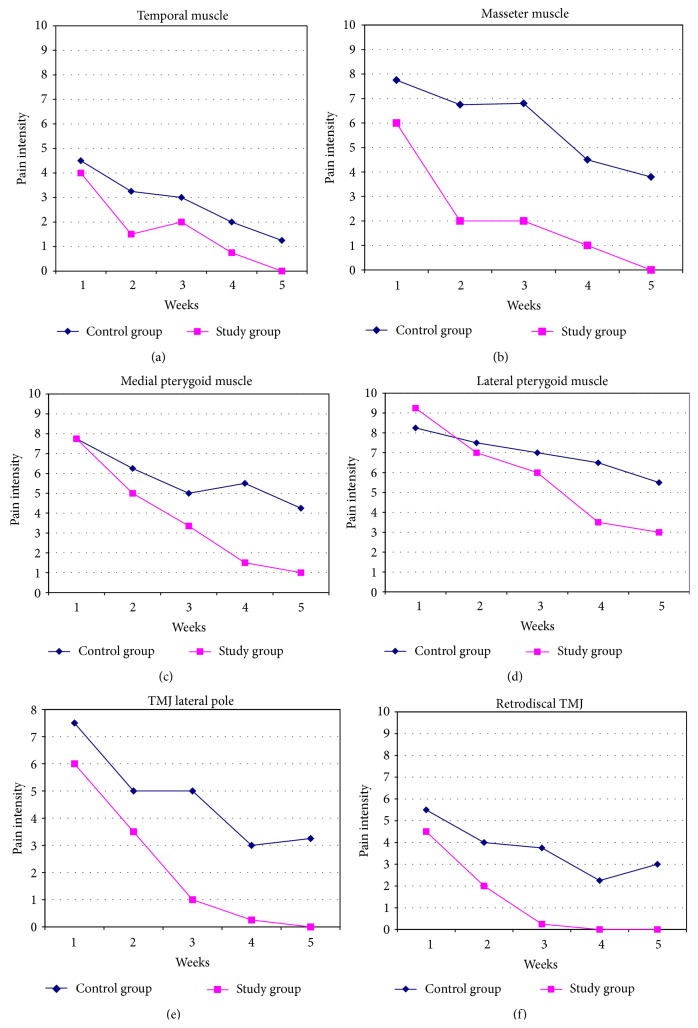
Pain intensity evolution under palpation exam of orofacial muscles and TMJ.

The data obtained by the EVA and subjected to statistical analysis showed the evolution of painful symptoms in TMD patients in five different moments of the evaluation survey and were represented by the average pain intensity assessed according to Figure 4, with regard to orofacial structures: temporal muscles (a), masseter (b), medial pterygoid (c), lateral pterygoid (d), TMJ (e), and retrodiscal TMJ (f). The evolution of symptoms in both groups assessed showed a statistically significant decrease (*p* < 0.05) in muscle and joint symptoms during treatment in both established therapeutic modalities (Table 3). However, in the first week of therapy, the intensity of pain on palpation proved to be lower in the experimental group, for most of the structures evaluated (Table 4).

The Mann-Whitney test allowed a statistical comparison between groups and showed that pain intensity values were equivalent (*p* > 0.05) at first (T1) and statistically different (*p* < 0.05) at the end of the evaluation (T5). There was a significant reduction in symptoms in the study group, in comparison with the control group from the second assessment (except for temporal muscles in T3 and lateral pterygoid in T2, Table 4).

## 4. Discussions

Literature reports TMD prevalence in women at working age of about 83.7% [2, 7]. Therefore, this study selected such individuals in order to have a homogeneous sample. The subjects studied showed joint and muscle TMJ with pain intensity equal to or higher than 4.0, indicated by VAS.

Diagnosis of energy pattern according to TCM indicated the most prevalent pathologies:* Kidney Yin *deficiency,* Spleen Yang* deficiency, and* Liver Yang *ascension. Such standards are common in muscle TMD patients [30–32]. However, even with similar orofacial symptoms, TMD patients have different eastern diagnostics and etiology when classified by* Ba Gan Bian Zheng *[28].

The ear acupuncture therapy was done with the same acupoints for all experimental patients, in accordance with the quantitative methodology for controlled clinical trial [23]. Although each individual's need for energy may vary, we sought to select common acupoints that would act on the orofacial region and on organs and viscera systems, according to standardized acupuncture methodology [14, 23]. We considered the anatomical regions with painful manifestations, muscle regions, ligament, bone, and joint TMD, so as to justify the prescription of points:* Liver*,* Spleen*, and* Kidney*, acting on the orofacial areas [21]. The Maxillary and Jaw regions in the ear are suitable for this type of disease when there is an obstruction of* Qi* and* Xue* manifested by muscle or joint pain or by myofascial pain trigger points, which are commonly found in TMD patients [21, 30–32]. Therapeutic analgesia could also be obtained when the* Occipital* and* Shen Men* acupoints were influenced, with the reduction of pain through sedation of* Yang* hyperactivity, especially in the* Liver*, often manifested by symptoms of pulsatile headache, tinnitus, and vertigo [21].

Still, most of the patients have reported alterations in their emotional state before the therapy, possibly due to the interruption of the free flow of* Qi* and consequently of emotions or to the compromise of the* Shen*. For this reason, we have chosen the* Shen Men* and* Liver* acupoints to promote clearance and normalization of the free flow [21, 30–32].

It is rare to find serious adverse effects of the acupuncture treatment, but some of them might be the following: the pain from puncture, fatigue, and circulatory disorders [33]. During this study, none of the patients have reported any serious side effects, except for the feeling of* De Qi *and presence of heat and of the electrical stimulation itself, which occurred during the therapy. This information confirms the results of a project [33] conducted by the German Social Security, when it studied the adverse effects and complications of the acupuncture treatment in patients with chronic pain. They concluded that adverse effects are mild, which characterizes the therapy as safe.

Therapies associated with dental treatment are considered adjuvants [34]. These should not be used alone when the etiological condition is not controlled [1], as in bruxism. Our study shows that ear therapy associated with occlusal splint was more effective than when compared to splint therapy isolated. Patients in the experimental group showed significant reduction in pain symptoms in the first evaluation, which corroborates the result of studies carried out by some authors used as [25, 26]. This investigation is relevant as it demonstrates that adjuvant therapy for ear acupuncture induces faster improvement and stability of orofacial symptoms.

Literature review showed that some publications raise the possibility of aggregate placebo effect in acupuncture therapy [35]. Generally, they consider the effects produced as physiological mechanisms of analgesia promoted by acupuncture, but they do not consider the placebo action as an effect of this therapy exclusively [35]. Acupuncture is routinely used in veterinary medicine and pediatric clinics and it has shown favorable results, considering that these individuals are not influenced by suggestions of psychological nature [35]. Other studies conclude that acupuncture has promoted a great reduction in pain if compared to placebo [5, 24].

We are not aware of any other study in the literature that uses a similar methodology or ear acupuncture needle for TMD pain remission. However, as well as traditional acupuncture, this therapy was able to promote greater analgesia in the experimental group, which corroborates other studies [21].

The results of the occlusal splint therapy alone in this research were also plausible for muscle painful reduction, as well as the study that evaluated the pain threshold evolution in the masseter and temporalis muscles using occlusal splints [36]. However, in another clinical trial [26], joint pain did not improve when patients were submitted to occlusal therapy. The results of the present clinical study demonstrate that the acupuncture analgesic effect was able to act even on joint areas. The significant pain reduction in the palpation of the lateral pole in the experimental group was probably due to the analgesic action of acupoints that influence muscles and joint areas.

The limitations of this research involve mainly the small number of patients, the need for long-term monitoring, the comparison with a placebo control group, and a posttreatment evaluation. However, the use of strict criteria for inclusion and exclusion and the difficulties in the application of sham acupuncture are realities that should be considered. Furthermore, it was difficult to have patients with pain agreeing to participate in studies for extended periods.

Changes in the stomatognathic system, especially those caused by pain, have been the object of studies in dentistry due to their multifactorial and complex etiology [1–3, 9]. The search for a better quality of life, with chronic symptom reduction, justifies the adoption of adjuvant therapies such as acupuncture [15, 16]. The modality of ear acupuncture microsystem is scarcely referenced in scientific literature. However, the results of this research showed its effectiveness in the control of pain and optimization of the results of occlusal therapy, by demonstrating synergistic action with the conventional dentistry treatments, resulting in lower levels of pain intensity in the short term.

Pain improvement results in better quality of life. With faster and more expressive results, there are better adherence to treatment, better prognosis, and better physical and psychological responses for patients. This can be explained by analgesia results provided by acupuncture's action on central nervous system neurotransmitters (western view) or by restoring the flow of* Qi* and* Xue* in channels and collaterals (eastern view).

## 5. Conclusion

According to the results, we have concluded that, in short-term treatment, ear acupuncture adjunct therapy has reduced pain symptoms of muscle and joint TMD, more rapidly and more significantly than isolated occlusal therapy.

## Figures and Tables

**Table 1:** Distribution of volunteers in each group, baseline.

	Patient	Age (years, month)	Symptoms duration (months)	VAS baseline (main complaint)	Rdc/tmd diagnosis		
					Group I Muscle disorders	Group II Disc displacement	Group III Other conditions
SG	1	32, 2	10	8	I.a, BIL	II.a UNI	III.a
	2	18, 4	14	8	I.a BIL	—	III.a
	3	24, 4	9	9	I.b BIL	—	III.a
	4	54, 8	20	7	I.a UNI	II.a UNI	III.b
	5	38, 2	12	10	I.a UNI	II.a BIL	III.b
	6	58, 3	14	9	I.b UNI	II.c UNI	III.b
	7	49, 7	12	9	I.a BIL	—	III.b
	8	30, 0	15	9	I.b BIL	—	III.a
	9	27, 9	18	7	I.B BIL	II.a UNI	III.a
	10	42, 1	18	7	I.a BIL	II.a UNI	III.a

CG	11	26, 2	8	6	I.b BIL	II.a UNI	III.a
	12	36, 1	22	10	I.b UNI	II.a UNI, II.bUNI	III.a
	13	24, 4	10	8	I.b UNI	—	III.a
	14	48, 1	12	8	I.b UNI	II.a UNI	III.a
	15	55, 10	18	9	I.a BIL	II.a UNI	III.b
	16	51, 0	15	8	I.a UNI	II.a UNI	III.a
	17	34, 2	9	8	I.a UNI	—	III.a
	18	38, 2	18	10	I.a BIL	—	III.a
	19	45, 4	15	9	I.a BIL	II.a UNI	III.b
	20	44, 4	8	8	I.b BIL	II.a BIL	III.a

VAS: Visual Analog Scale; UNI: unilateral; BIL: bilateral.

**Table 2:** TCM pathology and symptoms in the study individuals.

Pathology default	Symptoms	Individuals (*n*)
*Liver Yang* hyperactivity	Headache, tinnitus, irritability, heat, feeling thirsty Pulse in rope, red and fluttering tongue	6

*Spleen Yang* deficiency	Tiredness, lethargy, repetitious thoughts, bruxism Empty pulse pale and flaccid tongue	4

*Kidney Yin* deficiency	Tiredness, heat, unrest, bruxism, headache, red malar Fine and fast pulse, red tongue, without coating	3

*Liver Qi* stagnation	Depression, abdominal distention, sigh, headache Pulse in rope, violet tongue	3

Liver and biliary vesicle humidity and heat	Bitter taste, jaundice, dizziness, fatty food difficulty, temporal headache, stress Slippery pulse, yellow and coat tongue	2

*Kidney Jing* deficiency	Weak memory and concentration, tinnitus, arthralgia, insecurity Deep and fine pulse, fine and flaccid tongue	2

**Table 3:** Evaluation of pain evolution between time points assessed.

Pain intensity variation palpation						
	Temporal muscle		Masseter muscle		Medial pterygoid muscle	
	Control group	Study group	Control group	Study group	Control group	Study group
*T*1 × *T*2	n.s.	n.s.	n.s.	0.036	n.s.	0.047
*T*1 × *T*3	n.s.	0.017	n.s.	0.031	n.s.	0.046
*T*1 × *T*4	0.023	0.046	n.s.	0.031	n.s.	0.038
*T*1 × *T*5	0.002	0.001	n.s.	0.018	n.s.	0.030

	Lateral pterygoid		Lateral pole of TMJ		Retrodiscal TMJ	
	Control group	Study group	Control group	Study group	Control group	Study group

*T*1 × *T*2	n.s.	0.037	n.s.	n.s.	n.s.	0.040
*T*1 × *T*3	n.s.	0.029	n.s.	0.032	n.s.	0.031
*T*1 × *T*4	n.s.	0.033	n.s.	0.012	n.s.	0.043
*T*1 × *T*5	n.s.	0.015	n.s.	0.032	n.s.	0.040

*T*: time; value of significance: *p* < 0.05; n.s.: not significant; Friedman statistical test.

**Table 4:** Evaluation of intergroups to average of intensity pain in each time point assessed.

	*T*1	*T*2	*T*3	*T*4	*T*5
Control group × study group					
Temporal muscle	n.s.	0.042	n.s.	0.039	0.38
Masseter	n.s.	0.003	0.012	0.008	0.011
Medial pterygoid	n.s.	0.044	0.025	0.005	0.015
Lateral pterygoid	n.s.	n.s.	0.045	0.024	0.020
Lateral pole of TMJ	n.s.	0.047	0.003	0.016	0.009
Retrodiscal TMJ	n.s.	0.039	0.045	0.006	0.017

*T*: time; value of significance: *p* < 0.05; n.s.: not significant; Friedman statistical test.
